# Recent Advances in Short QT Syndrome

**DOI:** 10.3389/fcvm.2018.00149

**Published:** 2018-10-29

**Authors:** Oscar Campuzano, Georgia Sarquella-Brugada, Sergi Cesar, Elena Arbelo, Josep Brugada, Ramon Brugada

**Affiliations:** ^1^Medical Science Department, University of Girona, Girona, Spain; ^2^Cardiovascular Genetics Center, IDIBGI, Girona, Spain; ^3^Centro Investigación Biomédica Red Enfermedades Cardiovasculares, Madrid, Spain; ^4^Pediatric Arrhythmia Unit, Cardiology Department, Hospital Sant Joan de Déu Barcelona, Barcelona, Spain; ^5^Institut Clínic Cardiovascular, Hospital Clínic, Universitat de Barcelona, Barcelona, Spain; ^6^Institut d'Investigacions Biomèdiques August Pi i Sunyer, Barcelona, Spain; ^7^Familial Cardiomyopathies Unit, Hospital Universitari de Girona Doctor Josep Trueta, Girona, Spain

**Keywords:** sudden cardiac death, arrhythmias, short QT syndrome, genetics, QT interval variability

## Abstract

Short QT syndrome is a highly malignant inherited cardiac disease characterized by ventricular tachyarrhythmias leading to syncope and sudden cardiac death. It is responsible of lethal episodes in young people, mainly infants. International guidelines establish diagnostic criteria with the presence of a QTc ≤ 340 ms in the electrocardiogram despite clinical diagnostic values remain controversial. In last years, clinical diagnosis, risk stratification as well as preventive therapies have been improved due to identification of pathophysiological mechanisms. The only effective option is implantation of a defibrillator despite Quinidine may be at times an effective option. Currently, a limited number of rare variants have been identified in seven genes, which account for nearly 20–30% of families. However, some of these variants are associated with phenotypes showing a shorter QT interval but no conclusive diagnosis of Short QT syndrome. Therefore, an exhaustive interpretation of each variant and a close genotype-phenotype correlation is necessary before clinical translation. Here, we review the main clinical and genetic hallmarks of this rare entity.

## Introduction

In 2000, a new cardiac channelopathy was described in one family with history of sudden cardiac death (SCD) (1). All relatives analyzed showed idiopathic persistent short QT interval in the electrocardiogram (ECG); one of them also showed paroxysmal atrial fibrillation (AF). Due to its main feature in the ECG, it was called Short QT Syndrome (SQTS). In 2003, two additional unrelated families were described, with mutiple relatives across several generations showing history of SCD. Several relatives showed the same ECG previously reported 3 years before, complaining from palpitations, and syncope; this fact highlighted the familial nature of this novel disease and the malignant arrhythmogenic events associated (2). Currently, more than 350 publications (PubMed: short QT syndrome) have been reported but a low number of cases (almost 250 cases) and families (nearly 150 families) have been diagnosed worldwide; therefore it is classified as a rare disease. This lethal condition may be underdiagnosed because some affected individuals never experience symptoms. In most cases, highly malignant arrhythmias and a lethal outcome is identified, mainly at young age. Nowadays, a reduced number of genes has been associated with the disease. Hence, a comprehensive genetic testing has a diagnostic yield of nearly 20–30% (3). The short QT interval on the ECG is due to an accelerated cardiac repolarization (and shorter refractory periods) serving as substrate for ventricular arrhythmias leading to syncope and even SCD, sometimes the first manifestation of the disease.

## Clinical findings

### Prevalence

The limited number of cases wordwide difficult establish the real prevalence in global population. However, recent studies suggest a prevalence between 0.02 and 0.1% in adults while in pediatric population the prevalence is 0.05% (4). Arrhyhtmogenic events associated with SQTS have been recorded in all ages from infants to 80-year-old patients, but the first year of life appears to be the most alarming with a 4% rate of cardiac arrest (5). The probability of a first syncope, even SCD, is nearly 40% by 40 years old. One-third of cases with SCD as their first manifestation, and up to 80% of cases showed a personal or family history of SCD (6). Lethal events may occur in both genders, but a slight male predominance seems to exist, although no conclusive data exist concerning this point. A recent study suggests that male predominance may be due to higher testosterone levels and genes located on the X chromosome could be involved in QTc interval regulation (7).

### Clinical assessment

Clinical manifestations associated with SQTS may range from asymptomatic (up to 40% of cases) to dizziness, AF, ventricular arrhythmias, syncope and even SCD. Clinical manifestations can be particularly severe, especially in children, and may cause SCD in infants (sudden infant death syndrome—SIDS). As abovementioned, it is a familial disease; therefore, diagnostic patients usually have a family history of syncope or SCD in a young first or second-degree relative. Recent studies suggest that there are two high-risk peaks of SCD: in the first year of life and from 20 to 40 years old (8). Consequently, clinical assessment is recommended in all family members. Asymptomatic patients who carry a pathogenic variant associated with the disease are also at high risk because the first manifestation of the disease could be the SCD (9).

### Diagnosis

**Figure 1:**
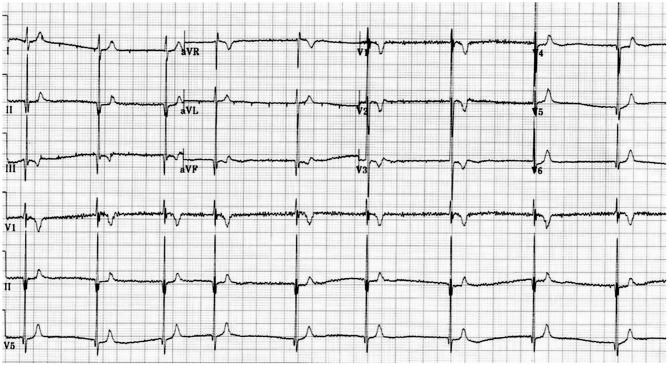
Electrocardiogram showing a short QT interval.

**Figure 2:**
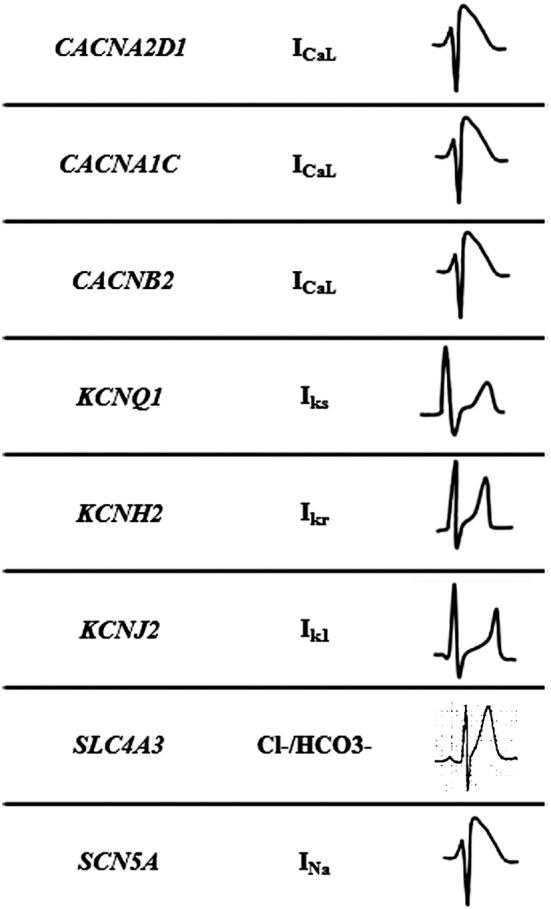
Mechanisms of Short QT syndrome. Gain-of-function mutations of potassium and loss of- function mutations of calcium and sodium channels result in an abbreviated repolarisation phase during action potential and shortening of the QT interval.

The main problem in clinical diagnosis is the definition of cut off value at the lower end of the QTc. In 2011 (10) and 2013 (11), guidelines/consensus documents defined the SQTS as: “*a genetic arrhythmogenic disorder characterized by a short and uniform QT/QTc intervals (*<*330 ms) on the ECG, with absent or minimal ST segments, with an interval from J point to T wave peak (Jp-Tp) measured in the precordial lead with the T wave of greatest amplitude*<*120 ms, possible tall T waves with narrow base similar to the T wave of moderate hyperkalemia (“desert tent T waves”), frequent early repolarization pattern, prolongation of T peak-T end interval, and possible presence of prominent U waves in the absence of structural heart disease and others disturbances that cause repolarization abnormalities*” (Figures 1, 2). In addition to a short QT interval, Tülümen et al. suggested the PQ segment depression as a novel marker for SQTS (12) despite further studies should be performed in order to clarify this point. Current guidelines (13) suggest the following diagnostic criteria:

1. QTc ≤ 340 ms (Class IC); or.
2. QTc ≤ 360 ms; and one or more of the following: (a) A confirmed pathogenic mutation. (b) Family history of SQTS.

(c) Family history of sudden death at 40 years of age. (d) Survival from a VT/VF episode in the absence of heart diseases (Class IIaC).

Resting 12-lead ECG should be performed at a heart rate within normal limits when the diagnosis of SQTS is suspected (14). Recent studies suggest that a QT/HR relationship slope under —0.9 ms/beat/min in the exercise test could be a useful tool in order to distinguish affected subjects from healthy individuals (15). Authors showed that the QT/HR slope is significantly flatter in the subgroup of males who carry a pathogenic variant in the *KCNH2* gene, which shows the shortest QT intervals, as compared with the subgroup with unknown genotype. In addition, the QT adaptation to standing is reduced, as compared to the values for a normal population. It has been reported that tissue Doppler imaging (TDI) and speckle tracking echocardiography (STE), could be part of the clinical assessment because of systolic function may also be affected and patients presented a dispersion of contraction in myocardium (16). In contrast, invasive electrophysiological study (EPS) with programmed ventricular stimulation is not recommended for SCD risk stratification (13).

### Risk stratification and treatment

Due to the low number of patients with SQTS, risk stratification represents indeed the main current challenge in clinical characterization. Electrophysiological testing has not been useful in predicting cardiac arrest but patients showing QTc intervals < 340 ms should be considered at highest risk of SCD despite no conclusive results have been published, so far. The only predictor of cardiac arrest in a patient with SQTS found so far has been a previous history of cardiac arrest. Therefore, the optimum strategy for primary prevention of cardiac arrest in these patients is still a matter of argue. Nowadays, current guidelines recommend an implantable cardiac defibrillator (ICD) as the first and more effective therapeutic measure in patients who have experienced sustained VT/VF episodes or survivors of an aborted cardiac arrest (17). Inappropriate shock is a common complication in patients carrying an ICD. In asymptomatic patients showing a SQTS, due to lack of definite studies, the implantation or not of an ICD remains to be clarified (18). Recent reports suggest that an ICD might be considered in SQTS patients with a strong family history of SCD and previous evidence of short QTc. However, an alternative therapy is necessary, especially in small children and in adults in whom ICD cannot be a therapeutical option.

Therefore, an alternative treatment to ICD is the pharmacological approach. In last years, several drugs such as Ibutilide, Flecainide, Sotalol, Disopyramide, Nifekalant, Propafenone, Carvedilol, Metoprolol, and Amiodarone have been used but none have undergone conclusive clinical studies, primarily due to the lack of families as wel as low event rate in SQTS patients. Nowadays, SQTS patients are treated with administration of Sotalol or Quinidine, which prolong cardiac repolarization and consequently QT interval due to inhibition of repolarization (19). Sotalol is ineffective in patients carrying pathogenic variants in the *KCNH2* gene (SQTS type 1), the most common gene associated with SQTS. Quinidine, which has similar affinity to the open and inactivated states of IKr, is effective therapy for type 1 SQTS (20). Unfortenatelly, Quinidine has been removed from the market in several countries and often has intolerable side-effects. In a recent study, treatment with Hydroxyquinidine was associated with a lower incidence of arrhythmic events in SQTS patients as it prolongs the QT interval (5). Pharmacological treatment in symptomatic patients is recommended, particularly if ICD is not implanted, and in asymptomatic patients with a family history of SCD.

### Genetic basis

This malignant entity can have a congenital origin or acquired. The first genetic alteration associated with the disease was reported in 2004, located in the *KCNH2* gene (21). Currently, more than 30 rare variants have been identified in 8 genes (*CACNA1C, CACNA2D1, CACNB2, KCNH2, KCNJ2, KCNQ1, SCN5A*, and *SLC4A3*), and follow an autosomal dominant pattern of inheritance with high phenotype penetrance (Table 1). SQTS is associated with gain-of-function alterations in genes encoding outward K^+^ channels and loss-of-function mutations in genes encoding different subunits of cardiac L-type Ca^2+^ channel (22). A reduction in inward repolarizing currents and/or an increase in outward repolarizing currents will favor early repolarization, leading to action potential duration (APD) shortening (reduced QT interval). It predisposes to reentrant mechanisms, which can lead to AF and VF (23).

**Table 1:** Genes associated with Short QT Syndrome or Shorter than normal QT interval.

**Gene**	**Protein**	**Phenotype**	**Prevalence**
*CACNA2D1* *CACNA1C* *CACNB2*	Calcium Voltage-Gated Channel Auxiliary Subunit α2/δ1 Calcium Voltage-Gated Channel Subunit Alpha1 C (Cav1.2) Calcium Voltage-Gated Channel Auxiliary Subunit Beta 2 (CavB2)	BrS + Short QT interval BrS + Short QT interval BrS + Short QT interval	<1% <1% <1%
*KCNQ1*	Potassium Voltage-Gated Channel Subfamily Q Member 1 (Kv7.1 or Kv1.9)	SQTS	<5%
*KCNH2*	Potassium Voltage-Gated Channel Subfamily H Member 2 (hERG or Kv11.1)	SQTS	15%
*KCNJ2*	Potassium Voltage-Gated Channel Subfamily J Member 2 (Kv2.1 or Kir2.1)	SQTS	<5%
*SLC4A3*	Solute Carrier Family 4 Member 3	SQTS	<1%
*SCN5A*	Sodium channel, voltage gated, type V α subunit (Nav1.5)	BrS + Short QT interval	<1%

*BrS, Brugada Syndrome; SQTS, Short QT Syndrome*.

Rare variants identified in genes encoding potassium channels (*KCNH2, KCNJ2, KCNQ1*) and the *SLC4A3* gene, have been associated with SQTS. In contrast, rare variants located in genes encoding calcium (*CACNA1C, CACNA2D1*, and *CACNB2*) and the *SCN5A* gene, have been associated with Brugada syndrome (BrS) concomitant with shortened QT intervals, but without a conclusive diagnosis of SQTS (24). A comprehensive genetic analysis of all known genes identifies a potential damaging variant in nearly 30% of cases. However, this percentage may be misleading due to the low number of reported families (25). Current guidelines recommend a genetic analysis of only five genes (*KCNH2, KCNQ1, KCNJ2, CACNA1C*, and *CACNB2b*) in all clinically diagnosed or suspected SQTS cases due to high lethality (13).

The mechanism of arrhythmogenesis in SQTS is not well understood. Several pre-clinical studies focused on unravel the pathofisiological mechanism involved in SQTS have been performed. Most part of these studies are *in vitro* analysis of potential pathogenic variants. Additionally, *in vivo* approaches, mainly transgenic animal models, have been also performed focused on selected variants identified in families showing highly malignat phenotypes, even SCD, in most part of relatives. In last years, *in silico* approaches have been also performed, using algorythms reproducing biological systems similar to SQTS (26). Despite these molecular advances, of nearly 30 potential pathogenic variants associated with SQTS, only a slight number can be classified as definitely pathogenic following recent recommendations of American College of Medical Genetics and Genomics and the Association for Molecular Pathology (ACMG/AMP) (27). In recent years, it has been developed the induced pluripotent stem cell–derived cardiomyocytes (hiPSC-CMs) which allow a most exhaustive study of cellular mechanism of diseases. In 2018, and for the first time, it has been generated a hiPSC-CMs model from a SQTS patient carrying a pathogenic variant in the *KCNH2* gene. Patient-specific hiPSC-CMs are able to recapitulate single-cell phenotype features of SQTS and provide novel opportunities to further elucidate the cellular disease mechanism and test drug effects (28).

### Potassium channels

**Figure 3:**
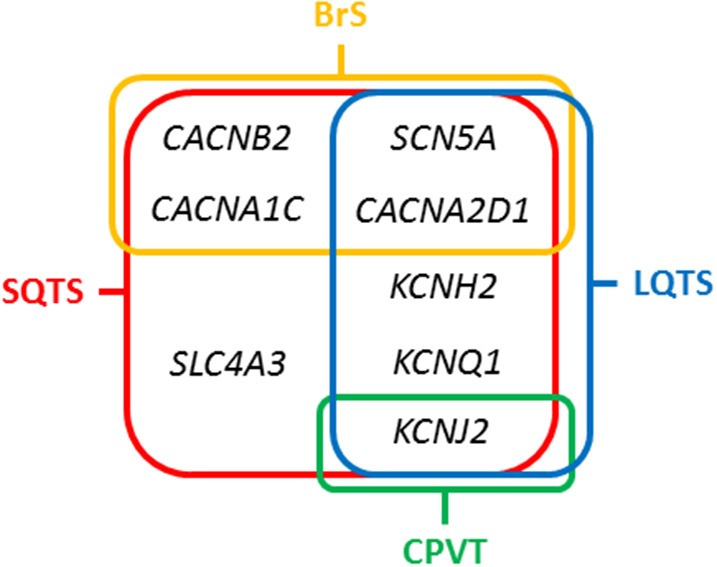
Overlapping genes in Short QT Syndrome. LQTS, Long QT Syndrome; BrS, Brugada Syndrome; CPVT, Catecholaminergic Polymorphic Ventricular Tachycardia.

The main gene is *KCNH2* (ID: 3757) which encodes a voltage-activated potassium channel belonging to the ether-a-go-go (EAG) family-potassium voltage-gated channel, subfamily H (EAG-related), member 2 (Kv 11.1 α subunit/hERG). It mediates the rapidly activating component of the delayed rectifying potassium current in heart (IKr) (29). It is the main gene, associated with the so-called SQTS type 1, and responsible of 15% of all cases. Generally, cardiac events are associated with adrenergic in situations such as noise or exercise, but it may also occur at rest. In 2017, Hu et al. performed an exhaustive study of the phenotypic and functional expression of the highly frequent rare variant associated with SQTS worldwide (30). The hotspot variant (p.T618I) causes a major gain of function in IKr, leading to acceleration of repolarization, which underlies the abbreviation of the QT interval. Other seven rare variants have been also reported in SQTS and associated with SQTS with a potential pathogenic role -p.N588K (c.1764C>A), N588K (c.1764C>G), p.I560T, p.E50D, p.W927G, p.R1135H, and p.R164C- despite further studies should be done in order to clarify their definite role in SQTS. This gene is also associated with other cardiac channelopathies, mainly Long QT syndrome (LQTS), and BrS (Figure 3). Therefore, genetic interpretation of variants in *KCNH2* should be done with caution despite most part of current reported variants seems to play a deleterious role. The second gene associated with SQTS is *KCNQ1* (ID: 3784). It encodes a voltage-gated potassium channel (Kv7.1 α subunit) required for repolarization. This protein can form complexes associated with MinK (the *KCNE1* gene) and MiRP2 (the *KCNE3* gene), both also potassium channel proteins. When it is associated with KCNE1, forms the (IKs current) cardiac potassium current and induces a rapid activation of potassium-selective outward current. The Kv7.1 protein may be also associated with MiRP2 protein to form the potassium channel (31). It is associated with the so-called SQTS type 2, and responsible of nearly 5% of all cases. Currently, five rare variants has been identified potentially associated with SQTS (p.Phe279Ile, p.Val307Leu, p.Val141Met, p.Ile274Val, and p.Arg259His). This gene is also associated with other cardiac channelopathies, mainly LQTS (Figure 3). The third potassium gene associated with SQTS is *KCNJ2* (ID: 37591). This gene encodes an integral membrane protein and inward-rectifier type potassium channel (Kir2.1 α subunit). Inward rectifier potassium channels are characterized by a greater tendency to allow potassium to flow into the cell rather than out of it (IK_1_ current). It is associated with the so-called SQTS type 3, and responsible of nearly 5% of all cases (32). Currently, four rare variants has been identified potentially associated with SQTS (p.Asp172Asn, p.Glu299Val, p.Met301Lys, and p.Lys346Thr). This gene has been also associated with other channelopathies, mainly Catecholaminergic Polymorphic Ventricular Tachycardia (CPVT; Figure 3).

### Calcium channels

Concerning calcium channels, the main gene is *CACNA1C* (ID: 775). This gene encodes an alpha-1 subunit of a voltage-dependent calcium channel (calcium channel, voltage-dependent, L type, alpha 1C subunit) (Cav1.2 α subunit). All variants identified so far decrease inward currents at early phases of cell repolarization (I_Ca,L_), induce transmural and epicardial dispersion of repolarization leading to a combined phenotype of BrS and short QTc interval. It is associated with the so-called SQTS type 4, and responsible of < 1% of all cases (22). Currently, 10 rare variants have been potentially associated with SQTS despite further studies should be done in order to confirm their certain pathogenic role (p.Ala39Val, p.Gly490Arg, p.Asn547Ser, p.Arg632Arg, p.Glu1115Lys, p.Arg1780His, p.E1829_Q1833dup, p.Arg1880Gln, p.Val2014Ile, and p.Asp2130Asn). This gene has been also associated with other channelopathies, mainly LQTS (Figure 3). The second calcium gene is *CACNB2* (ID: 783). This gene encodes a subunit of a voltage-dependent calcium channel protein, a member of the voltage-gated calcium channel superfamily (Cav1.2 β subunit). The beta subunit of voltage-dependent calcium channels contributes to the calcium channel function by increasing peak calcium current, shifting the voltage dependencies of activation and inactivation, modulating G protein inhibition and controlling the alpha-1 subunit membrane targeting. Nowadays, only on rare variant has been associated with SQTS (p.Ser481Leu). As also occurs in the *CACNA1C* gene, variants identified in *CACNB2* were associated with BrS and shortened QT interval. It is associated with the so-called SQTS type 5, and responsible of < 1% of all cases (22). This gene has been also associated with other channelopathies, mainly LQTS (Figure 3). The third calcium gene is *CACNA2D1* (ID: 781). It encodes a member of the alpha-2/delta subunit family, a protein in the voltage-dependent calcium channel complex (Ca_v_1.2 α2/δ1 subunit). The protein regulates calcium current density and activation/inactivation kinetics of the calcium channel (I_Ca,L_). Only one rare variant has been reported in this gene associated with SQTS (p.Ser755Thr). No conclusive data exist about the association of this gene and SQTS. Therefore, additional studies should be performed in order to clarify a conclusive association. It is associated with the so-called SQTS type 6, and responsible of < 1% of all cases (22). This gene has been also associated with other channelopathies, mainly LQTS (Figure 3).

### Sodium channel

In 2012, it was identified the p.R689H variant in the *SCN5A* gene (ID:6331) (33). This gene encodes the sodium channel protein type 5-subunit alpha (Nav1.5) which mediates the voltage-dependent sodium ion permeability of myocite membranes. This gene has been also associated with other familial channelopathies, mainly BrS (Figure 3). Concerning SQTS, the reported patient was an asymptomatic 40-year-old male with family history of SD of unknown origin who had a Brugada-like ECG with short QT intervals. This variant has been identified in global databases despite in low frequencies (ExAC: 0.011% and gnomAD: 0.01). Thererfore, no conclusive data exist concerning the association of this variant with SQTS. Despite this fact, it is so-called SQTS type 7, and potentially responsible of < 1% of all cases. At our point of view, genetic translation of *SCN5A* variants in SQTS patients should be done with caution due to its ambiguous role.

### The *SLC4A3* gene

In 2017, the *SLC4A3* gene (ID:6508) was associated with SQTS (34). This gene (Solute Carrier Family 4 Member 3) encodes a plasma membrane anion exchange protein 3 (AE3). It mediates a part of the Cl-/HCO3- exchange in cardiac myocytes. To date, only one rare variant (p.R370H) have been identified in the *SLC4A3* gene associated with SQTS. It follows an autosomal dominant pattern of inheritance. The pathogenic variant leads to a trafficking defect, decreased Cl,HCO3-exchange over the cell membrane and increased intracellular pH, shortened the APD and it reduces QT interval. This variant has not been identified in global databases, reinforcing its potential deleterious role. However, no conclusive data exist concerning the association of this variant with SQTS. It is associated with the so-called SQTS type 8, and responsible, so far, for < 1% of all cases (Figure 3). At our point of view, genetic translation of variants in this gene should be done with caution due to its ambiguous role in SQTS.

## Conclusions

Nearly 20 years ago, SQTS was reported as a familial arrhyhtmogenic entity. Nowadays, low number of families have been reported but with a high lethality. This lack of families impedes the stablishment of a conclusive risk stratification scale, particulary in asymptomatic cases carrying a genetic alteration. New development of hiPSC-CMs from patients may allow unraveling pathophysiological mechanism, helping to understand or treat the disease. Patients suffering of SQTS are at high risk of syncope and SCD. Implantation of an ICD remains the most effective preventive measure after aborted SCD and malignant ventricular arrhythmia although pharmacological therapies may be used in certain cases, especially in children. Currently, nevertheless improving advances in genetics, almost 70–80% of families remain without a genetic cause identified after a comprehensive analysis. Genotype-phenotype analysis are necessary in order to improve current guidelines in early identification as well as prevention in families suffering of SQTS.

## Author contributions

All authors listed have made a substantial, direct and intellectual contribution to the work, and approved it for publication.

### Conflict of interest statement

The authors declare that the research was conducted in the absence of any commercial or financial relationships that could be construed as a potential conflict of interest.
